# Chromatin landscape associated with sexual differentiation in a UV sex determination system

**DOI:** 10.1093/nar/gkac145

**Published:** 2022-03-07

**Authors:** Josselin Gueno, Michael Borg, Simon Bourdareau, Guillaume Cossard, Olivier Godfroy, Agnieszka Lipinska, Leila Tirichine, J Mark Cock, Susana M Coelho

**Affiliations:** Sorbonne Université, UPMC Univ Paris 06, CNRS, UMR 8227, Integrative Biology of Marine Models, Station Biologique de Roscoff, CS 90074, F-29688 Roscoff, France; Department of Algal Development and Evolution, Max Planck Institute for Biology Tübingen 72076, Tübingen, Germany; Sorbonne Université, UPMC Univ Paris 06, CNRS, UMR 8227, Integrative Biology of Marine Models, Station Biologique de Roscoff, CS 90074, F-29688 Roscoff, France; Sorbonne Université, UPMC Univ Paris 06, CNRS, UMR 8227, Integrative Biology of Marine Models, Station Biologique de Roscoff, CS 90074, F-29688 Roscoff, France; Sorbonne Université, UPMC Univ Paris 06, CNRS, UMR 8227, Integrative Biology of Marine Models, Station Biologique de Roscoff, CS 90074, F-29688 Roscoff, France; Sorbonne Université, UPMC Univ Paris 06, CNRS, UMR 8227, Integrative Biology of Marine Models, Station Biologique de Roscoff, CS 90074, F-29688 Roscoff, France; Department of Algal Development and Evolution, Max Planck Institute for Biology Tübingen 72076, Tübingen, Germany; Nantes Universite, CNRS, US2B, UMR 6286, F-44000, Nantes, France; Sorbonne Université, UPMC Univ Paris 06, CNRS, UMR 8227, Integrative Biology of Marine Models, Station Biologique de Roscoff, CS 90074, F-29688 Roscoff, France; Sorbonne Université, UPMC Univ Paris 06, CNRS, UMR 8227, Integrative Biology of Marine Models, Station Biologique de Roscoff, CS 90074, F-29688 Roscoff, France; Department of Algal Development and Evolution, Max Planck Institute for Biology Tübingen 72076, Tübingen, Germany

## Abstract

In many eukaryotes, such as dioicous mosses and many algae, sex is determined by UV sex chromosomes and is expressed during the haploid phase of the life cycle. In these species, the male and female developmental programs are initiated by the presence of the U- or V-specific regions of the sex chromosomes but, as in XY and ZW systems, sexual differentiation is largely driven by autosomal sex-biased gene expression. The mechanisms underlying the regulation of sex-biased expression of genes during sexual differentiation remain elusive. Here, we investigated the extent and nature of epigenomic changes associated with UV sexual differentiation in the brown alga *Ectocarpus*, a model UV system. Six histone modifications were quantified in near-isogenic lines, leading to the identification of 16 chromatin signatures across the genome. Chromatin signatures correlated with levels of gene expression and histone PTMs changes in males versus females occurred preferentially at genes involved in sex-specific pathways. Despite the absence of chromosome scale dosage compensation and the fact that UV sex chromosomes recombine across most of their length, the chromatin landscape of these chromosomes was remarkably different to that of autosomes. Hotspots of evolutionary young genes in the pseudoautosomal regions appear to drive the exceptional chromatin features of UV sex chromosomes.

## INTRODUCTION

In species that reproduce sexually, sex is often determined by a pair of sex chromosomes: X and Y chromosomes in male-heterogametic species, Z and W in female-heterogametic species or U and V in haploid sexual systems (1). Sex chromosomes originate from pairs of autosomes but further differentiate after the sex-specific chromosome (Y, W or both the V and U) stops recombining (1–3). Males and females have distinct sex chromosome sets but the extensive phenotypic differences between males and females (sexual dimorphism) are largely caused by differences in autosomal gene expression or so-called sex-biased gene expression. The nature and extent of sex-biased gene expression have been investigated in recent years across a broad range of taxa using genome-wide transcriptional profiling. These studies have revealed that sex-biased gene expression is common in many species, although its extent may vary greatly among tissues or developmental stages (4).

Although many reports have described the nature and evolution of sex-biased genes across several taxa, the molecular mechanisms underlying the regulation of sex-biased genes during sexual differentiation remain poorly understood. One prevalent mechanism to regulate gene expression is through covalent modifications such as DNA methylation and post-translational modification (PTMs) of histone tails. DNA methylation regulates transcription in diverse eukaryotes (5) and may contribute to transcriptional differences between sexes (6), playing for instance an important role in differentiating female morphs like workers and queens in the honeybee (7). In the liverwort *Marchantia*, male and female gametes have different levels of DNA methylation and this is correlated with differences in the expression of genes involved in DNA methylation (8). Histone PTMs are another important component of transcriptional regulation and can impact gene expression by altering chromatin structure or recruiting histone modifiers. Specific combinations of histone PTMs (so-called chromatin states) are associated with functionally distinct regions of the genome such as heterochromatic regions and regions of either permissive transcription or repression (9). The role of chromatin states in regulating gene expression patterns during development in animals and plants is well established [e.g. (10,11)]. However, few studies have carried out chromatin profiling during sexual differentiation to determine how chromatin is associated with sex-biased gene expression. In *Drosophila*, the genome-wide distribution of both active and repressive chromatin states differed between males and females, but sex-specific chromatin states appeared not to explain sex-biased expression of genes (12), although differences in the chromatin landscape of males and females influenced by the Y chromosome may contribute to sex-biased gene expression (13). Yen and Kellis (2015) (14) used a comparative epigenomic approach to contrast male versus female human samples, revealing that the X chromosome underlies epigenetic differences between sexes, but epigenomic differences are not reflected in gene expression differences. In the tunicate *Oikopleura dioica*, distinct combinations of histone PTMs were uncovered in testis versus ovaries (15).

In organisms with XY or ZW sex determination systems, sex chromosomes often exhibit unique patterns of gene expression and unusual patterns of chromatin marks compared with autosomes [e.g. (8,12,16,17)]. For instance, in *Drosophila* males, where the Y chromosome is transcriptionally repressed and the X chromosome is hyper-transcribed due to dosage compensation (18), both of these transcriptional modifications are correlated with changes in the chromatin configuration (19–22). Sex chromosomes are derived from autosomes, but they are governed by unique evolutionary and functional constraints (23,24). The sex-limited chromosome (Y or W) degenerates, i.e. loses most of its ancestral gene content, accumulates repetitive DNA and evolves a heterochromatic appearance (16,17,25,26). In contrast, the homologous chromosome (X or Z) acquires dosage compensatory mechanisms by evolving a hyper-transcriptional state (dosage compensation) (27–30). In *Drosophila*, the ratio of euchromatin-to-heterochromatin is different between the two sexes, which is mainly due to the presence of the a repeat-rich Y chromosome in males (12,13,31). Similarly, the Z-specific region in schistosomes has a unique chromatin landscape, dominated by active histone PTMs, that are associated with dosage compensation (32).

In contrast, little is known about how chromatin impacts sexual differentiation in organisms with a UV sexual system such as mosses and algae (33–38), although recent work has analyzed the patterns of histone post-translational modifications during the haploid-diploid life cycle of the brown alga *Ectocarpus* (39). In UV sexual systems, sex is expressed during the haploid phase of the life cycle, where inheritance of a U or V sex chromosome at meiosis determines whether the multicellular adult will be female or male, respectively (1,40). UV sexual systems differ markedly from XY and ZW systems (3,40–42). For example, sexual individuals will only have a single U or V sex chromosome, so chromosome-scale dosage compensation or meiotic sex chromosome inactivation mechanisms are unlikely to exist. Moreover, because Y or W sex chromosomes often undergo genetic degeneration, their size, repeat content and gene density is markedly different to the partner X or Z chromosome. In contrast, U and V chromosomes are expected to undergo only mild degeneration (35,41,43) and do not exhibit such an asymmetry because each chromosome functions independently in a haploid context and therefore experiences similar evolutionary pressures (43).

The brown alga *Ectocarpus* has emerged as a powerful model organism to study UV sexual systems (reviewed in 40). A reference genome is available for this species (44–46), including high quality assembly of its sex chromosomes (43,44,46–48). The *Ectocarpus* non-recombining U and V specific regions (SDRs) are relatively small compared with the pseudoautosomal regions (PAR) (the SDR occupies 1/10th of the sex chromosome), such that the large majority of the sex chromosome recombines within the PAR (43,47,48). Note that the female and male SDRs on the U and V chromosomes respectively have about the same physical size and share a similar number of, albeit distinct, genes (43). Given the lack of chromosome-scale dosage compensation, the small size of the SDR, and the fact that the SDRs displays only mild levels of degeneration, the U and V sex chromosomes in this system are not expected to present a markedly distinct chromatin landscape compared with autosomes. However, this prediction has never been tested.

The expression pattern of genes on the U and V sex chromosome differs from that of autosomal genes (40). For example, most *Ectocarpus* genes located on the U and V SDRs are upregulated in the haploid gametophyte phase of the life cycle (43,49). Moreover, when compared with autosomes, the sex chromosome PARs harbor an excess of evolutionary young or taxonomically restricted genes (48) and are enriched in both life cycle-related genes (sporophyte-biased genes) and female-biased genes (50). Nevertheless, what chromatin states associate with this intriguing composition of genes and patterns of gene expression in a UV sexual system still remains unclear.

Here, we investigated sex-specific chromatin landscapes of autosomes and sex chromosomes in *Ectocarpus*, a model brown alga with a UV sexual system. We built on our recent chromatin profiling in *Ectocarpus* by studying six different histone PTMs—four that are associated with gene activation, namely H3K4me3, H3K9ac, H3K27ac and H3K36me3, and two that are associated with reduced gene expression, namely H4K20me3 and H3K79me2 (39). H4K20me3 was associated with repeated sequences and H3K79me2 with genomic regions that often extended over several genes (39). Note that *Ectocarpus* DNA is not methylated (45), so we have not analyzed this modification here. Moreover, *Ectocarpus* lacks polycomb complexes and the associated PTMs, including the repression-associated mark H3K27me3 (39). Similarly, H3K9me2/3 have been detected in *Ectocarpus* but at very low abundance (39). Consequently, none of these additional repression-associated methylation marks were analyzed in this study.

Comparison of the profiles of these six histone PTMs with transcriptomic data showed that chromatin states were predictive of transcript abundance. The chromatin landscapes across the genomes of males and females were similar, overall. However, the chromatin signatures of genes that exhibited sex-biased expression was markedly different in males and females indicating that histone modifications may play an important role in mediating sexual differentiation. Moreover, a substantial proportion of the PAR genes presented sex-specific chromatin patterns. The U and V sex chromosomes were found to have very distinct chromatin landscapes to autosomes, despite the absence of a requirement for chromosome-scale dosage compensation in *Ectocarpus* and the fact that the U and V chromosomes do not exhibit strong signs of genetic degeneration.

## MATERIALS AND METHODS

### Biological material

The near-isogenic male (Ec457) and female (Ec460) *Ectocarpus* lines (Supplementary Table S1) were generated by crossing brother and sister gametophytes for either five or six generations, respectively (43). The resulting male and female strains, therefore, had essentially identical genetic backgrounds apart from the non-recombining SDR (Supplementary Table S2). To verify the homogeneity in terms of genetic background, we used the male and female input DNA from the ChIP-seq experiments aligned to the *Ectocarpus* reference genome to assess SNP diversity. The SNPs for male and female samples were called with bcftools mpileup and filtered for minimal mapping quality (–minQ 30), depth of coverage (–minDP 10) and missing data (–max-missing 0.9). We found 2,862,827 valid sites out of which 2,995 were variants (either SNPs or INDELS), differing from the reference genome (the SDR regions were excluded). We next compared the distribution of variant sites between males and females. Only 121 of the 2,995 variant sites were segregated between sexes, which accounts for 0.004% of all sites. Given this very low level of female/male polymorphism, it is highly likely that any differences we observed between the two strains are due to the presence of the female and male SDRs. Furthermore, note that the level of genetic diversity within the SDRs (which represent 869,870 bp and 893,800 bp in the female and male, respectively) has been shown to be extremely low (47), as is the case for the non-recombining regions for animals and plants (51). Therefore, the results presented here are likely to be representative of any male and female strain of *Ectocarpus* species, although they are based on only a single male V chromosome and a single female U chromosome.

Male and female gametophytes were cultured until near-maturity for 13 days as previously described (52) at 13°C in autoclaved natural sea water supplemented with 300 μl/L Provasoli solution, with a light:dark cycle of 12:12 h (20 μmol photons.m^–2^.s^–1^) using daylight-type fluorescent tubes. Note that we used between 400 and 600 male and female haploid individual gametophytes in each replicate, although there was only one genotype per each sex. Ten individual gametophytes were grown in each Petri dish. The level of maturity of male and female individual gametophytes was assessed under the microscope to ensure synchrony in terms of developmental stage. All manipulations were performed in a laminar flow hood under sterile conditions.

### Comparisons of male and female transcriptomes using RNA-seq

RNA for transcriptome analysis was extracted from the same duplicate male and female cultures as were used for the ChIP-seq analysis (see above). For each sex, total RNA was extracted from a mix of 90 gametophytes each, using the Qiagen Mini kit (http://www.qiagen.com). RNA quality and quantity were assessed using an Agilent 2100 bioanalyzer, associated with Qubit2.0 Fluorometer using the Qubit RNA BR assay kit (Invitrogen, Life Technologies, Carlsbad, CA, USA), as described previously (49,50).

For each replicate sample, cDNA was synthesized using an oligo-dT primer. The cDNA was fragmented, cloned, and sequenced by Fasteris (CH-1228 Plan-les-Ouates, Switzerland) using an Illumina HiSeq 4000 set to generate 150 bp single-end reads. See Supplementary Table S1 for RNA-seq accession numbers.

Data quality was assessed using FastQC (http://www.bioinformatics.babraham.ac.uk/projects/fastqc; accessed May 2019). Reads were trimmed and filtered using Cutadapt (53) with a quality threshold of 33 (quality-cutoff) and a minimal size of 30 bp.

Filtered reads were mapped to version v2 of the *Ectocarpus* sp. 7 reference genome (46,54) using TopHat2 with the Bowtie2 aligner (55). More than 85% of the sequencing reads from each library could be mapped to the reference genome (Supplementary Table S1). Note that the reference genome is from a male strain but the female SDR scaffolds have been added. Consequently, male and female data were mapped to the same reference genome.

The mapped sequencing data were then processed with featureCounts (56) to obtain counts for sequencing reads mapped to genes. Gene expression levels were represented as transcripts per million (TPMs). Genes with expression values below the fifth percentile of all TPM values calculated per sample were considered not to be expressed. This resulted in a total of 18,462 genes that were considered to be expressed.

Differential expression analysis was performed with the DESeq2 package (Bioconductor) (57). Genes were considered to be male-biased or female-biased if they exhibited at least a 2-fold difference (fold change; FC) in expression between sexes with a false discovery rate (FDR) < 0.05. A list of the sex-biased genes can be found in Supplementary Table S5.

To calculate breadth of expression we employed the tissue-specificity index tau (58) using published expression data from nine tissues or stages of the life cycle (female and male immature and mature gametophytes, mixed male and female gametophytes, partheno-sporophytes, upright partheno-sporophyte filaments, basal partheno-sporophyte filaments, diploid sporophytes) from *Ectocarpus* (46,48–50,59). This allowed us to define broadly expressed (housekeeping) genes (with tau<0.25) and narrowly expressed genes (tau>0.75).

### Genome-wide detection of histone PTMs

Male versus female *Ectocarpus* sp. gametophyte ChIP-seq experiments were carried for H3K4me3, H3K9ac, H3K27ac, H3K36me3, H4K20me3, and H3K79me2 and three controls (an input control corresponding to sonicated DNA, histone H3 and immunoglobulin G monoclonal rabbit (IgG)) as in (39). RNA-seq data (see above) was generated from the same samples, to ensure that the histone PTM and gene expression data were fully compatible. For ChIP-seq, 2.8 g (corresponding to 2,800 individual gametophytes) of *Ectocarpus* tissue was fixed for five minutes in seawater containing 1% formaldehyde and the formaldehyde eliminated by rapid filtering followed by incubation in PBS containing 400 mM glycine. Nuclei were isolated by grinding in liquid nitrogen and in a Tenbroeck Potter in nuclei isolation buffer (0.1% triton X-100, 125 mM sorbitol, 20 mM potassium citrate, 30 mM MgCl_2_, 5 mM EDTA, 5 mM β-mercaptoethanol, 55 mM HEPES at pH 7.5 with complete ULTRA protease inhibitors), filtering through Miracloth and then washing the precipitated nuclei in nuclei isolation buffer with and then without triton X-100. Chromatin was fragmented by sonicating the purified nuclei in nuclei lysis buffer (10 mM EDTA, 1% SDS, 50 mM Tris-HCl at pH 8 with cOmplete ULTRA protease inhibitors) in a Covaris M220 Focused-ultrasonicator (duty 25%, peak power 75, cycles/burst 200, duration 900 s at 6°C). The chromatin was incubated with an anti-histone PTM antibody (anti-H4K20me3, reference 5737S, anti-H3K4me3, reference 9751S and anti-H3K9ac, reference 9649S, Cell Signal Technology; anti-H3K27ac, reference 07360, Millipore; anti-H3K36me3, reference 9050, Abcam; anti-H3K79me2, reference D15E8, Cell Signal Technology) overnight at 4°C and the immunoprecipitation carried out using Dynabeads protein A and Dynabeads protein G. Following immunoprecipitation and washing, a reverse cross-linking step was carried out by incubating for at least 6 h at 65°C in 200 mM NaCl and the samples were then digested with Proteinase K and RNAse A. Purified DNA was analyzed on an Illumina HiSeq 4000 platform with a single-end sequencing primer over 50 cycles and pair-end sequencing for H3K79me2. At least 20 million reads were generated for each immunoprecipitation. The ChIP-seq dataset has been deposited in the NCBI Gene Expression Omnibus database under the accession numbers described in Table S2.

Quality control of the sequence data was carried out using FastQC (http://www.bioinformatics.babraham.ac.uk/projects/fastqc/). Poor quality sequences were removed and the high quality sequences trimmed with Cutadapt (53,60). Illumina reads were mapped onto the *Ectocarpus* v2 genome (46) using Bowtie (61), which contains both male and female SDR. Duplicates were removed using samtools markdup in the Samtools package (v 1.9) (62).

Quality control of ChIP-seq data sets followed the Encode ChIP-seq guidelines and practices (63) (Supplementary Table S3). ChIP-seq analysis was carried out for two biological replicates for each PTM in both the male and female samples. Spearman correlation analysis of replicates was performed with multiBamSummary and then by plotCorrelation (v3.1.2 deepTools) (64). Replicate samples were strongly correlated (Pearson correlations >0.92, Supplementary Figure S2).

To identify peaks and regions of chromatin mark enrichment in a gene-by-gene basis, each data set, after combining data for biological replicates, was analyzed separately for the male and female gametophyte. Peaks corresponding to regions enriched in H3K4me3, H3K9ac and H3K27ac were identified using the MACS2 (version 2.1.1) callpeak module (minimum FDR of 0.01) (65). H3K36me3, H3K79me2 and H4K20me3 were analyzed using SICER (v1.1) (minimum FDR of 0.01) (66,67) with a window size of 200 bp and a gap size of 400 bp. Note that peaks associated with sex-biased, PAR and SDR genes were manually inspected to validate reproducibility between replicates. The signal was normalized using the Signal Extraction Scaling (SES) method (68).

Heatmaps, average tag graphs and coverage tracks were plotted using EaSeq (69). Chord diagrams were generated using the circlize package in R (70).

### Detection of chromatin states and signatures

The six chromatin mark data sets were analyzed using ChromHMM (71) to learn a hidden Markov model and to assign chromatin states across the *Ectocarpus* genome. The bam alignment files for the six histone marks were converted into bed files with the software bedtools, option bamtobed (72). Then, ChromHMM was run using the « BinarizeBed» fonction on bed files with 200 bp per bin. A single joint model was learned using data from both male and female *Ectocarpus* and using the « LearnModel» function. We started with a 17-state model and then used the ChromHMM CompareModels module to compare decreasing numbers of states to the 16-state model. We then calculated for each of the 17 states the similarity (correlation between emission parameters) to its closest state in smaller models. A 12-state model was chosen as a point after which any further decrease in the number of states in the model resulted in states from the 17-state model being recovered with decreasing similarity.

The coverage of chromatin states in different categories of the genome was generated via the output files of ChromHMM called « Segmentation file ». An intersectBed (bedtools software) was made between states and the coordinates of all genes, allowing us to know which states overlap which genes.

**Figure 1.**
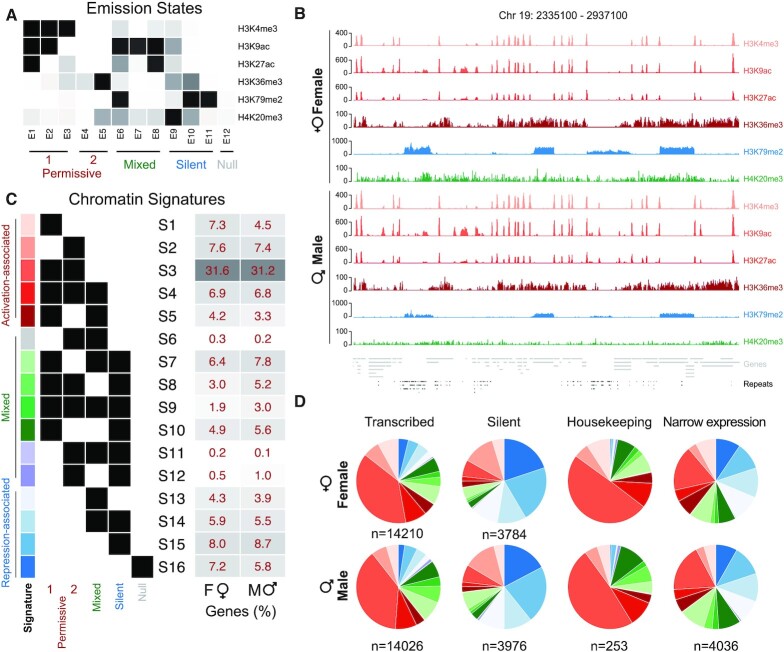
Histone PTMs and chromatin signatures of female and male *Ectocarpus*. (**A**) A model of prevalent chromatin emission states found in *Ectocarpus* using ChromHMM. Permissive 1 and Permissive 2, activation-associated states; Mixed, States that mix activation-associated and repression-associated chromatin PTMs; Silent, repression-associated chromatin states; Null, absence of assayed histone PTMs. (**B**) Representative region of the chromosome 19 showing profiles of mapped ChIP-seq reads for the six histone PTMs in females and males. Coverage is represented as the ratio of IP DNA relative to H3 for H3K36me3, H4K20me3 and H3K79me2 and input for TSS marks (H3K4me3, H3K9ac, H3K27ac). (**C**) Chromatin signatures assigned to genes based on ChromHMM states (see methods). Percentages of the total gene set associated with each chromatin signature in males (M) and females (F) are shown to the right. (**D**) Proportions of transcribed (TPM≥1TPM), silent (TPM<1TPM), housekeeping (tau<0.25) and narrowly expressed genes (tau>0.75) associated with each chromatin signature in males and females.

Because each gene in the genome is composed of multiple emission states, we simplified our analysis by grouping similar states into five major categories based on the presence/absence of activation-associated and repression-associated marks – ‘Permissive 1’ for states with mainly activation-associated TSS marks, ‘Permissive 2’ for states with mainly activation-associated mark H3K36me3, ‘Silent’ for states enriched in H4K20me3 and/or H3K79me2 marks, ‘Mixed’ for states with a combination of activation-associated and repression-associated marks and ‘Null’ for absence of marks (Figure 1A). We then associated these five major categories with each gene in the *Ectocarpus* genome to assemble a series of unique combinations that we termed ‘chromatin signatures’. Genes were considered to be in the null signature (S16) only when associated with no other emission state but E12. This resulted in a total of 16 distinct chromatin signatures across the *Ectocarpus* genome (S1-S16) (Figure 1C).

### Coverage for each histone PTM

The coverage for each histone PTM per chromosome was calculated using bedtools coverage where the coverage of each PTM was normalized by the size of the chromosome. The pseudoautosomal regions (PAR) and the sex-specific, non-recombining regions (SDR) of the sex chromosome were analyzed separately, as in (12).

### Statistical analysis

Statistical analysis was performed in R 3.6.3. Permutation tests were performed to study the differences of proportions of chromatin states in PAR and SDR genes compared to autosomal genes. We randomly subsampled 100,000 times a number of chromatin states equal to the number of PAR genes, SDR genes or both, from autosomal genes in order to perform proportion tests. We compared observed and simulated Pearson’s Chi-square statistics to assess whether the observed differences in chromatin state proportions between gene sets (autosomal, SDR, PAR, SDR+PAR) were statistically due to chance. A significant *P*-value indicates that the observed difference in proportion is not due to chance. In order to eliminate any possible effect of transposable element (TEs) prevalence (which is different between PAR, SDR and autosomal genes) we also performed these tests using a randomized set of autosomal genes that displayed exact the same TE prevalence. Similarly, we performed permutation tests (100,000 permutations) to determine whether the distribution of chromatin states of evolutionary young genes was significantly different from that of evolutionary conserved autosomal genes with similar expression levels (within 25% of the median), separately in males and females. We then compared Pearson's Chi-square statistics between observed and simulated datasets.

We performed linear models of log2(TPM+1) as a function of chromatin signatures and the interaction between chromatin state and genomic location (i.e. autosome or PAR). We report significant *P*-values in bold when states significantly influence the level of expression (state S1 used as reference level). Interaction term is significant when the effect of chromatin state of expression level is significantly different for an autosomal gene compared with a PAR gene.

We used the list of evolutionary young genes identified in (48,59). In brief, evolutionary young genes, i.e. genes that are taxonomically restricted to *Ectocarpus*, were defined as genes present in the genome of only *Ectocarpus* and having no BLASTp match (10^−4^ e-value cutoff) with a range of other stramenopile genome-wide proteomes from public databases (indicating that they are likely to have evolved since the split from the most recent common ancestor): the brown algae *Cladosiphon okamuranus*, *Macrocystis pyrifera*, *Saccharina japonica*, *Scytosiphon lomentaria*, the eustigmatophyte *Nannochloropsis gaditana*, the pelagophyte *Aureococcus anophagefferene*, and the diatom *Thalassiosira pseudonana*.

### GO-term analysis

Gene set enrichment analysis (GSEA) was carried out separately for each sex, grouping genes with either activation-associated (S1-S5) or repression associated (S13-S16) signatures. We used Fisher’s exact Test implemented in the R package TopGO (R package version 2.38.1.) to identify significantly enriched terms in biological processes. The top 30 categories (*P*-value<0.01) were plotted using ggplot2 package for R.

## RESULTS

### Identification of chromatin states in males and females of *Ectocarpus*

Our previous work associated H3K4me3, H3K9ac and H3K27ac to the transcription start sites (TSS) of active genes, whereas H3K36me3 was associated with gene bodies (39). H4K20me3 was associated with repeated sequences, particularly transposons, whereas H3K79me2 peaks often covered several kilobases and included multiple genes. The presence of H3K79me2 and H4K20me3 on gene bodies correlated with decreased transcript abundance (39). Thus, H3K4me3, H3K9ac, H3K27ac and H3K36me3 may be considered activation-associated marks and H3K79me2 and H4K20me3 repression-associated marks in *Ectocarpus*. Together, these six histone PTMs are therefore expected to provide a broad overview of the chromatin landscape in male and female *Ectocarpus*.

Near-isogenic male and female gametophyte (haploid) lines (Supplementary Table S1 and Figure S1) were used to generate sex-specific ChIP-seq profiles for the six histone PTMs (Supplementary Tables S2–S3). The male and female haploid lines were generated by inbreeding over five and six generations respectively, and were virtually identical genetically except for the sex-specific region (SDR) of the sex chromosome (see Materials and Methods, and Supplementary Table S2). We profiled at least 400 individual, clonal gametophytes for each male and female replicate line and confirmed high reproducibility between our ChIP-seq replicates (Supplementary Figure S2).

We used the ChromHMM algorithm to define twelve representative chromatin states common to males and females based on distinct combinatorial patterns of the six histone PTMs (Figure 1A and Supplementary Figure S3). Emission states E1-E3 consisted of combinations of the TSS-enriched activation-associated marks H3K4me3, H3K9ac and H3K27ac (designated as group ‘permissive 1’) while emission states E4-S5 (group ‘permissive 2’) corresponded to regions enriched in HK36me3 (Figure 1A). Emission states E6-E8 corresponded to mixed states that all included H4K20me3 or H3K79me2 together with one or more of the activation-associated marks (group ‘mixed’). Finally, silent emission states (E9-E11) were all enriched with H3K79me2 and/or H4K20me3 and H3K36me3 (group ‘silent’) while emission state E12 corresponded to a ‘null’ state that was devoid of the histone PTMs we assayed (group ‘Null’). An example of the histone PTM coverage over a 602 kbp region of the *Ectocarpus* genome is shown in Figure 1B.

While ChromHMM provided a broad overview of chromatin states across the *Ectocarpus* genome, our aim was to focus on chromatin changes between females and males at the gene level. Because more than one emission state could be present over the length of a gene in a multitude of combinations, we determined which of the five above groups of emission states were represented at each gene in the *Ectocarpus* genome and then defined a total of 16 distinct combinations of these groups that we herein refer to as chromatin signatures (see Materials and Methods section) (Figure 1C). Based on the predominant histone PTMs represented in each signature, the signatures were then classed into three groups: activation-associated, mixed and repression-associated.

### Chromatin signatures of different categories of *Ectocarpus* genes

To elucidate the relationship between chromatin signatures and gene expression in *Ectocarpus*, we generated paired RNA-seq data using the same biological samples as those used for the ChIP-seq analysis (see Materials and Methods section). Together with previously published datasets (49,50,73), we defined four categories of genes based on their expression patterns: transcribed genes (TPM≥1), silent genes (TPM<1), housekeeping genes with broad expression patterns in multiple tissues and life cycle stages (tau < 0.25; see Materials and Methods) and narrowly expressed genes (NEGs; tau > 0.75; see Materials and Methods).

The most common chromatin signature for the transcribed genes (38.4% and 38.2% in females and males, respectively) was S3, which corresponds to co-localization of three or four of the activation-associated histone PTMs (H3K36me3, H3K27ac, H3K9ac, H3K4me3; Figure 1D, Supplementary Table S4). At ‘silent’ genes, signature S15 containing H3K79me2 and H4K20me3 was the most common (21.7% and 21.9% in females and males, respectively; Figure 1D, Supplementary Table S4). Consistent with their ubiquitous expression, the majority of the housekeeping genes in *Ectocarpus* (49% and 48.8% in males and females, respectively) were associated with activation-associated signature S3. In contrast, NEGs were associated with a much larger proportion of mixed and repression-associated signatures compared with housekeeping genes, consistent with the restricted expression of NEGs (Figure 1D and Supplementary Table S4). Finally, the relative proportion of chromatin signatures was broadly similar between males and females for each of the four gene categories (transcribed, silent, housekeeping and NEG) (Figure 1D and Supplementary Table S4). Together, these data support our categorisation of activation-associated or repression-associated chromatin at *Ectocarpus* genes across the genome and suggests that the chromatin landscape remains largely stable during sexual differentiation.

### Identification of histone PTMs associated with gene activation and gene repression

**Figure 2.**
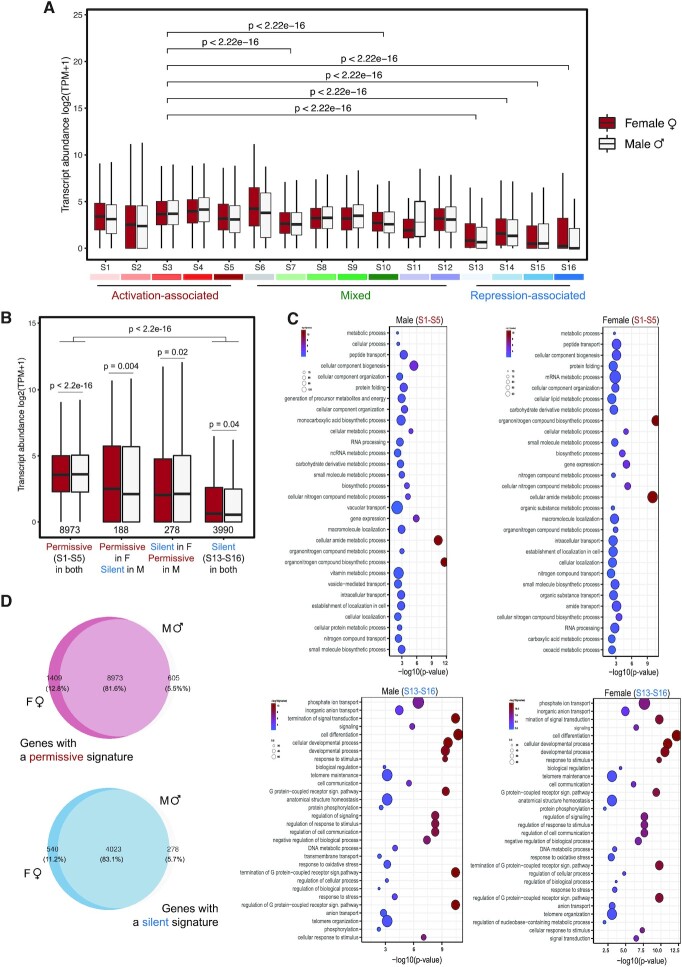
Gene expression and chromatin states. (**A**) Transcript abundances for genes associated with different chromatin signatures in males and females. The colour code is the same as that used in Figure 1A,B. Transcript abundances for genes exhibiting either activation-associated (S1-S5) or repression-associated (S13 or S16) chromatin signatures in females (dark pink) and males (gray). (**C**) GO term enrichment for genes marked with activation-associated or repression-associated chromatin signatures in males and females. (**D**) Venn diagrams representing the proportion of genes marked with activation-associated or repression-associated chromatin signatures in males and females.

To further investigate the relationship between the observed chromatin signatures and gene expression, we assessed the transcript abundances of genes corresponding to each chromatin signature. Consistently, genes that were assigned to an activation-associated signature had higher transcript levels than genes with mixed signatures, whereas genes assigned to a repression-associated signature exhibited the lowest levels of expression overall (Figure 2A). A clear trend toward increasingly higher transcript abundance was correlated with the gradual acquisition of more activation-associated marks (H3K9ac, H3K27ac, H3K4me3 and H3K36me3; Figure 2A; Supplementary Table S5). These observations support the proposed association of these four histone PTMs with gene activation (39) and further validate our assignment of chromatin signatures.

Conversely, genes corresponding to chromatin signatures with H4K20me3 and/or H3K79me2 consistently exhibited lower transcript levels than genes with equivalent chromatin states without H4K20me3 and H3K79me2 (Figure 2A and Supplementary Table S5). For example, transcript abundance for genes with signature S13 was significantly lower than genes with signature S3 (Wilcox test, *P*-value < 2.22E10-16 Figure 2A; Supplementary Table S5). These results are consistent with H4K20me3 and H3K79me2 being associated with repressed gene expression in *Ectocarpus*. Note however that because H4K20me3 is frequently associated with transposons (39), the observed association with reduced gene expression could also be indirect through the silencing of intronic transposon sequences. Finally, *Ectocarpus* genes associated with null signature S16, which corresponded to regions devoid of any of the assayed histone PTMs, exhibited very low transcript abundance (Figure 2A and Supplementary Table S5).

Next, we compared the expression level of genes with activation-associated signatures (S1-S5) in males and females to genes with repression-associated signatures (S13-S16). Note that we did not include genes with mixed signatures in this analysis because they exhibited a combination of both activation-associated and repression-associated marks and because they were expressed at intermediate levels (Figure 2A). As expected, genes marked with active signatures were expressed at higher levels in both sexes than those that were associated with repressive-associated signatures (Figure 2B; pair-wise Wilcox test, *P*-value<2.2E-16). Importantly, levels of gene expression in males and females were also significantly different for genes marked with active signatures in one sex but with repression-associated signatures in the other (Figure 2B; pair-wise Wilcox test, *P*-value = 0.004 and *P*-value = 0.02). Thus, despite the lack of global changes in chromatin landscape between males and females, our profiles illustrate localized chromatin signature changes associated with sex-specific gene expression.

Gene ontology (GO) term enrichment analysis showed that genes with activation-associated (S1-S5) signatures were enriched in functions related to metabolic process, whereas genes with repressive signatures (S13-S16) were enriched in functions related to signalling (Figure 2C). Interestingly, GO term enrichment appeared more stable between sexes with repressive-associated chromatin signatures, whereas sex-specific GO term enrichment was more apparent for genes with active signatures (Figure 2C). This difference was not due to repression-associated signatures being more stably maintained at genes in males and females compared to activation-associated signatures because similar proportions of genes exhibited stable maintenance of either activation-associated or repression-associated signatures in females versus males (83.1% and 81.6%; respectively; Figure 2D). Therefore, it appears that genes with activation-associated chromatin signatures exhibit more sex-specific functions than those with repressive signatures.

### Chromatin signatures and sex-biased gene expression in *Ectocarpus*

To investigate the role of histone PTMs in sexual differentiation, we examined the chromatin signatures of genes with sex-biased expression. A comparison of gene expression patterns in the two near-isogenic male and female lines (Supplementary Figure S1), based on RNA-seq data generated using the same biological samples as were used for the ChIP-seq analysis, identified a total of 268 genes that exhibited sex-biased expression (adjusted *P*-value < 0.05, fold change > 2, TPM > 1; Supplementary Tables S5 and S6).

The presence of the active signatures was associated with higher transcript abundance for sex-biased genes in both males and females (Supplementary Figures S4 and S5). Sex-biased genes therefore display a similar association between activation-associated chromatin and increased gene expression levels as that observed genome-wide (Figure 2A). Interestingly, 38.2% of male-biased genes (MBGs) and 37.5% of female-biased genes (FBGs) had a different chromatin signature between males and females (Supplementary Table S5), suggesting that chromatin dynamics underlie sex-biased gene expression in males and females.

**Figure 3.**
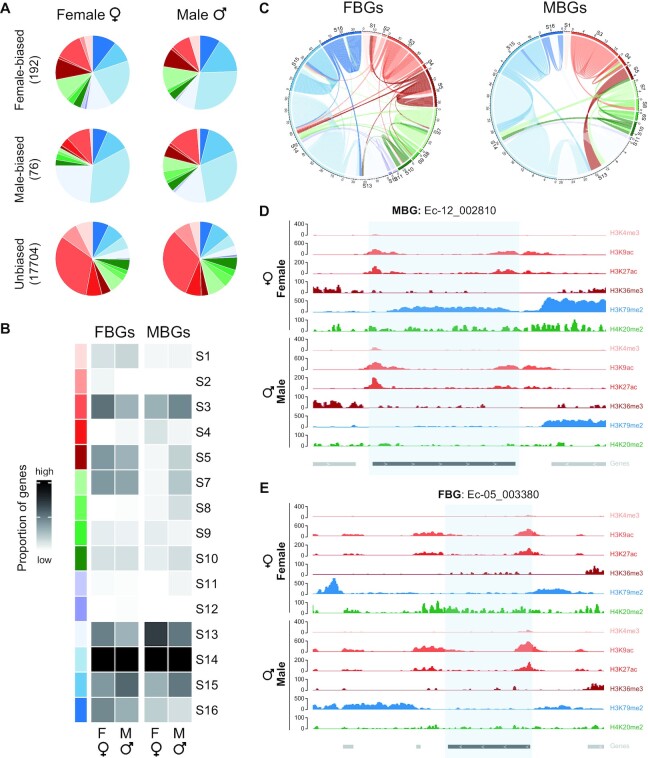
Histone PTM patterns at sex-biased genes in *Ectocarpus* males and females. (**A**) Proportions of the 16 chromatin signatures for female-biased, male-biased and unbiased genes in females (left) and males (right). The number of genes in each category are inside brackets. Chromatin signature color codes correspond to Figure 1C. (**B**) Proportions of genes associated with each of the 16 chromatin signatures for female-biased (FBG) and male-biased (MBG) genes in females (left) and males (right). The intensity of the gray squares is proportional to the number of genes corresponding to each signature. Colored squares represent the different chromatin signatures (see Figure 1A). (**C**) Chord diagrams comparing chromatin signatures associated with female-biased (left) and male-biased (right) genes in females and males. The color code for the chromatin states is the same as that used in Figure 1C. Each chord represents a sex-biased gene and illustrates whether a gene changes from one signature to another in the opposing sex. (**D**) Representative chromatin profiles for a male-biased gene on chromosome 12 in females and males. (**E**) Representative chromatin profiles for a female-biased gene in chromosome 5 in females and males.

Sex-biased genes tend to have narrow expression patterns (50,74) so we compared their chromatin patterns with that of NEGs. Overall, the proportions of the different chromatin signatures were significantly different compared with NEGs suggesting that their chromatin landscape is not related to their narrower pattern of expression (Chi-square test, *P*-value = 4.937E-15 and *P*-value = 0.01608 in FBGs versus NEGs in females and males, respectively, and *P*-value = 5.627E-4 and *P*-value = 3.333E-6 for MBGs versus NEGs in females and males, respectively; Figures 1D and 3A).

For the MBGs, there was a difference between the relative proportions of the different chromatin signatures in males compared to females. In males, repression-associated chromatin signatures were less frequent than in females, whereas signatures that included activation-associated marks (H3K9ac, H3K27ac, H3K4me3 and/or H3K36me3) were more common (Chi-square test *P*-value = 0.05; Figure 3A,B; Supplementary Table S4). A subset of MBGs (14.5%) changed from a repression-associated or mixed signature in females to an activation-associated signature in males (Figure 3C; Supplementary Table S7). A similar situation, albeit less clear, was observed for the FBGs, where activation-associated and mixed signatures were more frequent in females (47.4%) compared with males (38.9%), although not significantly (Chi-square test *P* = 0.093). Conversely, a larger proportion (61%) of female-biased genes were in a repression-associated configuration in males compared with females (52.6%; Figure 3A,B; Supplementary Table S4) but, again, not significantly (Chi-square test, *P* = 0.097). As for the MBGs, 12.5% of the FBGs had repression-associated or mixed signatures in males whilst the same genes become associated with activation-associated in females (Figure 3C and Supplementary Table S7).

Figure 3D,E shows genome browser tracks at representative FBG and MBG genes illustrating histone PTM changes during sexual differentiation. The MBG Ec12_002810, which encodes a conserved protein of unknown function, had an activation-associated chromatin signature in males (Supplementary Table S5), but accumulated both H3K79me2 and H3K20me3 in females where it also exhibited decreased expression (Figure 3D). Conversely, the reduced male expression of the FBG Ec-05_003380, which encodes a peroxidase enzyme, was associated with the augmentation of an H3K79me2 domain downstream of the gene (Figure 3E). This observation suggests that H3K79me2 might undergo differential deposition between males and females. Indeed, we noted that sex-specific domains of H3K79me2 were present at 12.1% (632) and 9.1% (457) of genes in males and females, respectively (Supplementary Table S8). However, the majority of these loci (97.3% and 97.2%) were not differentially transcribed between males and females, suggesting that H3K79me2 dynamics might have an indirect impact on sex-biased gene expression.

We also noticed more frequent sex-specific deposition of H3K36me3, H3K79me2 and H3K20me3 (Supplementary Table S8) compared with H3K27ac H3K4me3 and H3K9ac, suggesting that the former may drive the changes in chromatin signatures observed in males versus females.

In conclusion, our analysis revealed chromatin signatures modifications that were concomitant with changes in sex-biased gene expression between males and females, with MBGs undergoing more histone PTM transitions during sexual differentiation compared with FBGs.

### The chromatin landscape of the *Ectocarpus* sex chromosomes

In organisms with diploid sexual systems (XY or ZW), sex chromosomes exhibit different patterns of histone PTMs to autosomes (12,27,32,75,76). Given the nature of the *Ectocarpus* UV system, where most of the U and V sex chromosome recombines at the PAR, a markedly different chromatin landscape in sex chromosomes compared with autosomes is not expected. We investigated the chromatin signature of genes in the PAR, SDR and autosomes of *Ectocarpus* to test this hypothesis.

**Figure 4.**
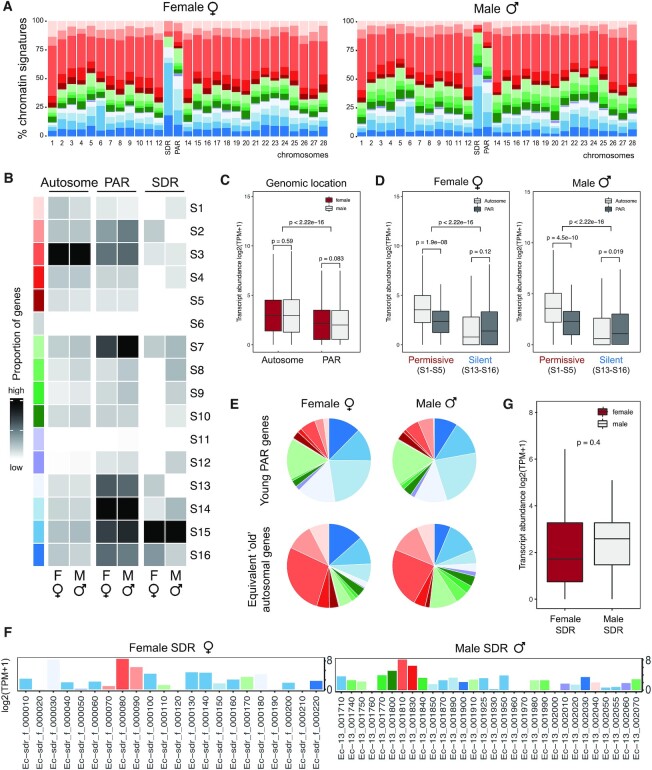
Chromatin landscape of the U and V sex chromosomes compared with the autosomes. (**A**) Chromatin signature distribution for each autosome and for the SDR and PAR regions of the sex chromosome in females (left panel) and in males (right panel). (**B**) Proportions of genes associated with each of the 16 chromatin signatures for all autosomes and for the PAR and SDR regions of the sex chromosome in females and in males. The intensity of the grey is proportional to the number of genes in each signature. The color code for the chromatin states is the same as that used in Figure 1A. (**C**) Transcript abundances, measured as log2(TPM+1), for autosomal and for PAR genes in males and females. Significant differences were assessed using pairwise Wilcoxon rank sum test. (**D**) Transcript abundances for autosomal and PAR genes associated with different chromatin signatures. Permissive, activation-associated signatures (S1-S5); Silent, repression-associated signatures (S13-S16). Significant differences were assessed using pairwise Wilcoxon rank sum test. (**E**) Chromatin state distribution of evolutionary young genes compared with autosomal conserved genes with similar expression patterns. See also Supplementary Table S15. (**F**) Transcript abundances, measured as log2(TPM+1), for individual genes located in the female and male sex determining regions (SDRs). Colored plots represent chromatin signatures corresponding to the color code indicated in Figure 1C (see also Supplementary Table S11). (**G**) Transcript abundances of genes located within the female and male sex-specific regions (SDRs). Significant differences were assessed using pairwise Wilcoxon rank sum test.

Surprisingly, a marked difference between sex chromosomes and autosomes was observed in *Ectocarpus* (Figure 4A, Supplementary Tables S5, S9–S12; Figures S7, S8, S9). While the relative proportion of the 16 chromatin signatures showed some variance between autosomes, genes on the sex chromosomes exhibited a strikingly different pattern compared to autosomal genes (Figure 4A). In particular, there was a significant under-representation of genes with activation-associated chromatin signatures (specifically S3 and S4) on the sex chromosomes compared to the autosomes (Supplementary Table S12). Furthermore, the sex chromosome was significantly enriched in signatures that included the histone PTMs H4K20me3 and H3K79me2 compared with autosomes (Figure 4A–C, Supplementary Table S12).

The significantly distinct chromatin patterns between the sex chromosome and the autosomes were also observed when only the PAR was taken into account (Chi-square test *P*-value <2.2E-16; Figure 4A,B). For example, 52.1% and 46.7% of the PAR genes in females and males, respectively, were associated with repressive signatures compared with 25.3% and 23.8% of autosomal genes for females and males, respectively (Supplementary Tables S5 and Table S10). Interestingly, 32% of the genes located in the PAR were found to be associated with different chromatin signatures in males and females (Supplementary Table S5), indicating that a substantial proportion of the PAR genes display sex-dependent chromatin signature transitions. Note that only 11 of the 430 PAR genes were classed as sex-biased genes (Supplementary Table S5), so these sex-related chromatin patterns on the PAR do not appear to be correlated with sex-biased PAR gene expression.

Analysis of the sex-determining regions of the U and V chromosomes showed that the vast majority (85%) of the genes within the female SDR (i.e. U-specific genes) were associated with repression-associated signatures, whereas this proportion was significantly less prevalent for the male SDR (where 53% of genes were associated with repressive signatures) (Chi-square test *P*-value = 0.02; Figure 4B; Supplementary Table S11). Therefore, male and female SDRs have distinct chromatin landscapes.

### Sex chromosome features and chromatin patterns

Previous work has shown that the *Ectocarpus* PAR is enriched in transposons compared with autosomes (43,48). Considering that H4K20me3 co-localizes with transposon sequences in *Ectocarpus* (39), we asked if the presence of transposons in PAR genes could explain the observed chromatin state distribution patterns. More PAR genes contained a transposon sequence compared to autosomal genes (80% versus 36%, respectively), but this did not correlate with an increased proportion of PAR genes marked with H4K20me3 (28–29% for the PAR versus 25–27% for autosomes) (Supplementary Table S13). Moreover, permutation tests using subsets of autosomal genes, in which 80% of the genes were selected to contain transposons (i.e., a similar proportion of genes with transposons to that observed for the PAR) indicated that the unusual pattern of chromatin signatures in the PAR was not due simply due to the presence of additional genes with transposon insertions (Supplementary Table S13).

Overall, transcript abundances for genes located in the PAR were significantly lower than for genes located on autosomes (Wilcoxon *P*-value < 2.22E-16; Supplementary Table S4, Figure 4C). This difference in expression level may potentially be explained by the different chromatin environment of the PAR and the autosomes. To test this hypothesis, we selected a subset of autosomal genes that had similar transcript levels to those of the PAR genes (Supplementary Table S14). Interestingly, the distribution of chromatin signatures for this set of autosomal genes was different to that of the PAR genes with similar expression levels (Figure 4E and Supplementary Figure S6) indicating that gene expression level was not the cause of the difference in chromatin signature patterns between the PAR genes and the autosomes. Moreover, the lower transcript abundance for PAR genes was consistent with a higher proportion of genes in repressive configurations compared with autosomal genes (52.9% and 46.7% for the PAR compared with 25.2% and 23.8% for the autosomes, in females and males, respectively; Supplementary Tables S10, S5). Note however that even PAR genes with an activation-associated chromatin signature had significantly lower expression levels compared to autosomal genes with similar signatures (pairwise Wilcoxon test, *P*-value = 1.9E-8, *P*-value = 4.5E-10 for females and males, respectively; Figure 4D). Thus, our results indicate that transcription level is not the sole cause for the striking chromatin differences between PAR genes and autosomal genes.

The PAR of *Ectocarpus* is enriched in evolutionarily young, *Ectocarpus*-restricted genes (‘young’ genes, see Materials and Methods section) (48). Young genes have unusual structural characteristics including shorter coding regions, fewer exons, lower expression levels and weaker codon bias compared with older genes (48,77,78). We thus asked if the presence of evolutionary young genes might explain the distinctive chromatin configuration in the PAR compared with autosomes. Genome-wide analysis of the 4,534 *Ectocarpus* young genes (59) revealed a significantly different distribution of chromatin signatures compared with more conserved genes with similar expression levels (Supplementary Table S15). Almost half of the PAR genes were classed as young genes (235 out of 440), which is a significant enrichment compared to autosomes (Chi-square test *P*-value<2.2E-16). Moreover, the distribution of chromatin signatures at young genes within the PAR was significantly different to that of conserved genes (Chi-square test, *P*-value = 1.57E-8 and *P*-value = 4.26E-11 in females and males, respectively) (Figure 4E, S6; Supplementary Table S10). When only evolutionarily conserved genes are included in the analysis the differences in chromatin distribution between the PAR and autosomes was considerably less marked (Supplementary Table S15). The enrichment of young genes on the PAR may therefore contribute to its unique chromatin distribution.

Taken together, our observations suggest that the sex chromosome exhibits significantly different features in terms of its chromatin landscape to the autosomes, not only at the level of the non-recombining SDR region but also for the PAR. The distinct chromatin features of the PAR are not explained by the preponderance of intragenic transposons nor by lower levels of gene expression but rather by the increased incidence of evolutionarily young genes.

### Chromatin signatures and expression of sex chromosome genes

Gene expression levels and deposition of chromatin marks were highly correlated for the complete set of *Ectocarpus* genes (see above, Figure 2A). For example, genes with an S3 signature enriched for all four activation-associated marks had significantly higher expression than genes with mixed signature S7 that was distinguished by the added presence of H3K79me2 and H4K20me3. However, when we analysed the association between expression level and chromatin state for genes located on the PAR of the sex chromosomes the situation was different. For example, the two chromatin signatures S4 and S6 in females had a significantly weaker correlation with expression on the PAR compared with autosomes. In males, a weaker correlation with gene expression was also observed for the PAR compared to the autosomes for signatures S4, S6, S7 and S12 (Supplementary Table S16 and Figure S10). In other words, depending on the location (PAR or autosomes) the correlation between chromatin signature and gene expression level was not the same.

With respect to SDR genes, the only significant correlation between expression level and chromatin signature was observed for chromatin signatures S3 and S4 in males and S15 and S16 in females (Supplementary Table S17), although the small sample size of SDR genes limited the power of the statistical test. Note that activation-associated mark H3K36me3 was more often present at male SDR genes (17/30) than at female SDR genes (4/22), with H3K36me3 coverage also higher on the male SDR than for the female SDR (Supplementary Figures S7, S8 and Table S9). We also noticed that median transcript levels for male SDR genes were higher than that of female SDR genes, although the difference was not significant (Figure 4G). In conclusion, our chromatin profiles suggest a different relationship between chromatin signature and expression levels for genes on the sex chromosomes compared with autosomes, further highlighting the unique chromatin configuration of UV sex chromosomes.

## DISCUSSION

### Chromatin regulation in a haploid UV sexual system

Three types of genetic sex determination system exist in nature: XX/XY, ZZ/ZW systems and U/V systems (1,33). For UV systems, studies have focused on understanding sex determination and sex-biased gene expression [e.g. (35,36,79)], but we know considerably less about chromatin patterns in males compared to females. Our study provides the first overview of sex-specific differences in chromatin landscape in a haploid UV system and its relationship with sex-biased gene expression, whilst also revealing the chromatin configuration of the U and V sex chromosome.

Analysis of six histone PTMs in *Ectocarpus* males and females resulted in the definition of 16 distinct chromatin signatures associated with genes. Chromatin signatures that included different combinations of H3K4me3, H3K9ac, H3K27ac and H3K36me3 were associated with transcriptionally active genes, whereas chromatin signatures that included H3K79me2 and/or H4K20me3 were associated with decrease gene expression compared to equivalent states lacking these marks. Signatures with H3K36me3 were associated with broadly expressed genes but were less prevalent on genes with narrow or tissue-specific expression, which could be related to a lower sensitivity in detecting H3K36me3 accumulation in a restricted subset of cells. The difference between housekeeping and NEG genes was considerably more marked for H3K36me3-containing signatures than for those with TSS-located marks (Supplementary Tables S4 and S5), perhaps indicating a stronger association of the former mark with gene transcription. A similar association of H3K36me3 with broadly expressed genes has been described for *Drosophila* (12,80), indicating that this correlation has been conserved across distantly related lineages. Overall, the *Ectocarpus* chromatin patterns described here are consistent with H3K4me3, H3K9ac, H3K27ac and H3K36me3 having similar roles in brown algae, land plants and animals (39,81–83). The role of H4K20me3, in contrast, appears to be less conserved across eukaryotic supergroups, being associated with low transcriptional levels in both animals and brown algae but with euchromatin and transcriptional activation in land plants (84,85).

Transcriptional reprogramming during life cycle transitions often involves the loss of repressive chromatin marks (86). Our recent profiling of the *Ectocarpus* sporophyte and gametophyte indicated that H3K79me2-enriched domains, which are associated with repressed genes in *Ectocarpus*, are stably maintained between generations (39). Interestingly, here we identified several examples of genes that were associated with H3K79me2 in one sex but not in another. However, the differential loss of this mark between sexes was not correlated systematically with changes in sex-biased gene expression, suggesting that H3K79me2 reprogramming might have only an indirect impact on transcription and sexual differentiation.

### Chromatin signatures of *Ectocarpus* sex-biased genes

When considered genome-wide, the proportion of genes associated with each chromatin signature did not differ substantially in males compared with females. However, when individual genes were compared, a considerable fraction was associated with different chromatin states in the two sexes, including genes that did not exhibit sex-biased expression patterns. Despite a lack of transcriptional changes in many cases, the strong correlation between chromatin signatures and gene expression argues that the chromatin reconfiguration we describe is biologically significant. One hypothesis is that the sex-specific alterations in chromatin manifest prior to any significant sex differences in transcription and phenotypic differentiation. In other words, differences in chromatin state may forecast sex-biased differences in gene expression during later stages of development, as reported for mammalian fetal germ cells (87). A more refined study using several stages during male and female gametophyte development would be needed to gain further insights into this matter.

In males, FBGs were more often associated with repression-associated signatures than in females. Similarly, more MBGs in females were marked with repression-associated signatures compared with males, whereas FBGs more had activation-associated or mixed signatures. About 37% of sex-biased genes had different chromatin signatures in males or females, which is significantly more than chromatin changes occurring at unbiased genes. These observations support a link, at least partial, between chromatin signature, expression pattern and role of sex-biased genes during sexual differentiation in *Ectocarpus*. Sex-specific chromatin states appear not to explain the sex-biased expression patterns in *Drosophila* (12) and mouse (88). It appears therefore that there is no absolute correlation between SBG and chromatin landscape in animals, and sex-biased gene expression may be regulated by other mechanisms, such as distal regulatory sites (88) or involving accessibility and 3D structure of chromatin (89). Future work focusing on these alternative mechanisms in *Ectocarpus* may help to further understand the regulation of sex-biased gene expression in the brown algae.

### Unique chromatin organisation features of the U and V sex chromosome

In organisms with UV sexual systems, the sex-specific SDRs of the U and V chromosome are both non-recombining, exhibit relatively similar structural features and appear to have been subjected to similar evolutionary pressures (43,79,90–93). In *Ectocarpus*, the SDR is relatively small, these chromosomes do not exhibit strong signs of degeneration and there is no chromosome-scale dosage compensation. Consequently, we did not expect the chromatin landscape of sex chromosomes to be substantially different to that of autosomes. Surprisingly, our results provided evidence that, contrary to this prediction, the *Ectocarpus* U and V sex chromosomes have a strikingly different chromatin environment to the autosomes.

Genes in the male SDR exhibited different patterns of chromatin signatures to genes in the female SDR, with H3K36me3 and H3K79me2 in particular being enriched on the male compared with the female SDR. Interestingly, H3K36me3 deposition is usually enriched on X chromosomes in animals where it plays a key role in dosage compensation (94). Deposition of H3K36me3 is known to be associated with increased transcript abundance in plants and animals (95,96), and, accordingly, we found that genes on the *Ectocarpus* male SDR exhibited higher expression levels than female SDR genes.

The *Ectocarpus* PAR has been shown to have unusual structural and gene expression features compared to autosomes (47,48), and we found here unusual patterns of chromatin signatures in this genomic region. This chromatin signature configuration is not explained by reduced gene expression levels in this region, nor by a greater prevalence of transposon insertions in PAR genes. Rather, our observations suggest that evolutionarily young genes, which are enriched in the PAR compared to autosomes, shape the chromatin environment of the sex chromosome. As has been observed for young genes in animals (97), evolutionary young genes in *Ectocarpus* exhibited markedly different chromatin patterns compared with evolutionary conserved genes. It is currently unclear why young genes are more abundant in the PAR compared with other genomic regions. One possible cause is the presence of higher amounts of transposons in the PARs, which may play a role in the emergence of new genes (77). This hypothesis is supported by the fact that young PAR genes often share homology with elements in the repeated fraction of the *Ectocarpus* genome (48).

Moreover, sex-specific differences in chromatin signature were prominent on the PAR of the U and V sex chromosome, where a large proportion of genes (32%) displayed different chromatin signatures between the two sexes. Our observations emphasize the unique features of the PAR of the *Ectocarpus* UV sex chromosomes compared to autosomes and suggest that transcription levels may depend on the genomic location of genes rather than solely the enrichment of histone PTMs, since the same chromatin signatures were transcribed differently on autosomes compared with sex chromosomes. It is possible that the expression of genes on the U and V sex chromosomes is regulated by different chromatin processes than those that regulate autosomal gene expression, perhaps involving histone PTMs we did not assay in this study. Note that the PAR features are unlikely to be caused by linkage disequilibrium with the SDR because the PAR is considerably large compared with the SDR and recombines extensively (48). Further investigation of the chromatin landscape of UV chromosomes in a diploid stage, and in several developmental stages during the haploid-diploid life cycle, together with further profiling in other species with a UV sexual system, promises to reveal more extraordinary features of these prevalent sex chromosomes.

## Supplementary Material

gkac145_Supplemental_Files
