# Supplementary material for: Chromatin landscape associated with sexual differentiation in a UV sex determination system
Source: Nucleic Acids Res. 2022 Mar 7;50(6):3307–22. doi: 10.1093/nar/gkac145 (PMC8989524; doi:10.1093/nar/gkac145)
Supplement: gkac145_Supplemental_Files [file gkac145_supplemental_files.zip › Supplemental Legends-Revision.docx]

## Supplemental Figures Legends

**Figure S1**. Pedigree of the male and female strains used in this study. SP, sporophyte; m, male gametophyte; f, female gametophyte.

**Figure S2**. Spearman correlation scores for comparisons of the genomic distributions of ChIP-seq signal peaks for the six histone PTMs. Rep1, replicate 1; Rep2, replicate 2.

**Figure S3**. Chromatin state annotation in males and females using ChromHMM.

**Figure S4**. Abundances of the transcripts of sex-biased genes (SBG) marked with different chromatin signatures in females and males. Abundances of transcripts of SBGs in different chromatin signatures in females (pink) and males (blue). Values in brackets indicate the number of genes analysed.

**Figure S5**. Abundances of transcripts of SBGs associated with each of the different chromatin signatures in males and females. The colour code is the same as that used in Figure 1A. The total number of SBGs associated with each signature are indicated in brackets.

**Figure S6**. Proportions of chromatin states for PAR genes compared with the proportions of chromatin states for a set of autosomal genes with a similar pattern of expression levels to the PAR genes.

**Figure S7**. Percentage of coverage for specific histone PTMs for the SDRs, PAR and autosomes in male and females. Scatter plot showing the percent of coverage (in base pairs) for each of the five histone PTMs, H3K4me3, H3K9ac, H3K27ac, H3K36me3, H3K79me2 and H4K20me3. Light blue and light pink represent coverage in male and female, respectively. Dark blue and red dots correspond to coverage for the V and U sex chromosomes, respectively. Light shading indicates the two PARs and dark shading the non-recombining, sex specific region (SDR) of the sex chromosome (chromosome 13).

**Figure S8**. Coverage (represented as percentage of base pairs) in three different genomic regions (PAR, SDR and autosomes) marked with different histone PTMs in females (left) and males (right).

**Figure S9**. Distribution of ChromHMM emission states across the sex chromosome and two representative autosomes. The colour code is the same as that used in Figure 1A.

**Figure S10**. Transcript abundances, measured as log2(TPM+1), for PAR genes associated with different chromatin signatures in males and females. The colour code is the same as that used in Figure 1C.

## Supplemental Tables Legends

**Table S1.** *Ectocarpus* strains used, RNA-seq sequencing statistics and SRA accession numbers.

**Table S2.** SNPs between male and female lines.

**Table S3**. Sequencing statistics for the ChIP-seq analysis and GEO reference for the dataset. N. peaks, number of peaks; FRiP, fraction of reads in peaks.

**Table S4**. Percentages of genes associated with each of the 16 chromatin signatures for different gene sets in males and females. Global, all genes in the genome; Transcribed genes, genes with TPM > 1; Silent genes, genes with TPM <1; Housekeeping and Narrowly-expressed, genes with tau <0.75 and tau >0.75, respectively; Unbiased, no sex-biased expression. For the chromatin signatures, refer to Figure 1A.

**Table S5**. Chromatin signatures (S1-S16) and transcript abundances (measured as TPM) for all *Ectocarpus* genes in males and females. FBG, female-biased gene; MBG, male-biased gene. For the chromatin states, refer to Figure 1C.

**Table S6.** Number of sex-biased genes in each of the chromatin signatures S1-S16 in males and females. FBG, female-biased gene, MBG, male-biased gene.

**Table S7.** Transitions between chromatin signatures observed for male-biased and female-biased genes in males compared with females. For chromatin signatures, refer to Figure 1C.

**Table S8**. Proportion of genes marked with each of the histone PTMs in males in females in different gene categories. Histone PTM peaks in each gene were analysed based on peak callers MACS2 and Sicer (see methods for details).

**Table S9.** Coverage of the six histone PTMs across male and female genomes. The sex chromosome (chromosome 13) is divided into PAR1 (pseudo-autosomal region 1), SDR (sex-determining region) and PAR2 (pseudo-autosomal region 2).

**Table S10.** Chromatin signatures of PAR genes in males and females.

**Table S11.** Chromatin signatures and transcript abundances (log2TPM+1) for SDR genes (see also Figure 4F).

**Table S12.** Permutation tests performed to determine whether the relative proportions of the different chromatin signatures were statistically different in different regions of the genome. We randomized the genomic location of autosomal genes 100,000 times and tested the difference between the observed proportions for the SDR, the PAR or the entire sex chromosome and the permuted gene sets using Chi-square statistics. Tests were performed independently for each chromatin state. Significant p-values (<0.01) are highlighted in bold.

**Table S13.** The presence of transposon sequences in the majority (80%) of PAR genes does not explain the distinct chromatin landscape of the PAR. Correlation between the presence of transposable elements within introns and the presence of H4K20me3 in PAR genes and autosomal genes (left table). Permutation tests comparing the proportion of each chromatin signature in the PAR with the proportion of that signature in 100,000 samples of 430 autosomal genes with transposon sequences in 80% of the genes. For most chromatin signatures, the proportion on the PAR was significantly different from those of the autosomal gene samples indicating that transposon content does not explain the unusual pattern of chromatin states observed for the PAR. Significant p-values (<0.01) are highlighted in bold (right table).

**Table S14.** Comparison of chromatin signatures of the PAR genes with those of a set of autosomal genes with a similar pattern of gene expression levels. To establish the autosomal gene set, for each PAR gene, the full set of autosomal genes was searched for the gene that had the most similar level of expression. When the TPM of the PAR gene was zero, an autosomal gene with a TPM of zero was selected at random. Figure S6 presents the proportions of chromatin signatures associated with the two gene sets.

**Table S15.** Permutation analysis to test whether there was a significant difference between the distribution of chromatin signatures in young genes compared with evolutionary conserved genes with similar expression levels (left table) and to test whether the distribution of signatures of PAR genes that are evolutionary conserved are different from autosomal genes (right table). Significant differences are highlighted in bold (p-value <0.01).

**Table S16.** Linear models to test whether there was a significant correlation between expression level (log2(TPM + 1)) and chromatin state (upper table) or to test whether location of a gene on the PAR or on an autosome significantly influenced the expression level associated with each chromatin signature (bottom table). Significant interaction terms, in bold, represent a significantly different effect of the chromatin signature on gene expression level in the PAR region compared to autosomal genes (p-value <0.05).

**Table S17.** Linear models to test whether there was a significant correlation between expression level (log2(TPM + 1)) and chromatin signature for SDR genes.
